# New‐onset bullous pemphigoid and flare of pre‐existing bullous pemphigoid after the third dose of the COVID‐19 vaccine

**DOI:** 10.1111/dth.15555

**Published:** 2022-05-11

**Authors:** Federico Bardazzi, Miriam Anna Carpanese, Diego Abbenante, Federica Filippi, Lidia Sacchelli, Camilla Loi

**Affiliations:** ^1^ Dermatology Unit IRCCS Azienda Ospedaliera‐Universitaria di Bologna Bologna Italy; ^2^ Division of Dermatology, Department of Experimental, Diagnostic and Specialty Medicine University of Bologna Bologna Italy

Dear Editor,

During the outbreak of coronavirus disease (COVID‐19), several cutaneous manifestations associated with COVID‐19 infection have been widely reported in literature. Currently, after the worldwide spread of a very large vaccination campaign, the occurrence of adverse events related to vaccinations has become the main interest of scientific research and reports.^1^ Cutaneous side effects to SARS‐CoV‐2 vaccination have been progressively reported so that many Journals have established a special section to collect them.

Here, we present a case series of four patients who developed bullous pemphigoid (BP) or worsened their pre‐existing and pharmacologically controlled BP after the third dose of the COVID‐19 vaccine (Table 1).

**TABLE 1:** Data of patients diagnosed with flare of pre‐existing BP or with new‐onset BP after the third dose of Covid‐19 vaccination

Sex, age	Bullous pemphigoid (PB) variant	Days after the third dose of vaccination	Anti‐BP 180 (U/ml)	Anti‐BP 230 (U/ml)	Vaccine	Therapy	Treatment outcome after 1 month
W/76	New‐onset PB	12	48	24	Pfizer‐BioNTech	Methylprednisolone initial dose of 0.5 mg/kg/die for 5 days and then gradually tapered + topical high potency steroids twice daily	Complete resolution
M/79	New‐onset PB	9	200	19	Pfizer‐BioNTech	Methylprednisolone initial dose of 0.5 mg/kg/die for 5 days and then gradually tapered + oral nicotinamide 750 mg/daily + topical high potency steroids once daily	Complete resolution
W/57	Flare of pre‐existing PB	7	10	3	Moderna	Topical high potency steroids twice daily for 15 days, then once daily until resolution	Complete resolution
M/62	Flare of pre‐existing PB	7	104	26	Pfizer‐BioNTech	Prednisone initial dose of 0.3 mg/kg/die for 7 days and then gradually tapered + topical steroids once daily	Complete resolution

Abbreviation: BP, bullous pemphigoid.

Two patients developed a new‐onset BP. The first patient was a 76‐years‐old woman with a history of leg ulcers, who developed a pruritic bullous eruption on the right leg and on the back, 12 days after her third dose of the Pfizer‐BioNTech COVID 19 vaccine. The second patient was a 79 years‐old‐man, coming from a nursing home, who developed bullous lesions on the trunk 9 days after the administration of the third dose of the Pfizer‐BioNTech COVID 19 vaccine. Both patients underwent skin biopsy; histopathology and direct immunofluorescence which confirmed the diagnosis of BP.

The other two patients were previously affected by BP, that was in clinical remission before the vaccination. The two patients, a 57‐years old woman and a 62‐years‐old man, had a flare of the pre‐existing disease, with pruritic and bullous skin eruption on the trunk and on the arms.

In both cases the symptoms occurred 7 days after the vaccination, respectively the Moderna and Pfizer‐BioNTech vaccine.

Although in these patients the clinical diagnosis was easily performed, in all of them we performed a biopsy for optic microscopy as well as direct immunofluorescence in order to confirm the diagnosis. In all of these patients the detection of anti BP‐180 and anti BP‐230 antibodies was performed, and the firsts were strongly positive.

All cases were treated with combination of topical steroids, nicotinamide, systemic steroids and their symptoms resolved within the 1‐month follow‐up.

Herein we report a case series of BP triggered by Covid 19 vaccine. BP is an autoimmune blistering disease that can be triggered by several factors, such as trauma, burns, drugs, and vaccinations^2^; in our cases, the temporal delay between the administration of vaccine and the appearance of blistering lesions suggests the association.

Skin adverse reaction to SARS‐CoV‐2 vaccination have been progressively reported. According to a recent review, two main categories of cutaneous manifestations caused by vaccination, which we both observed in our patients, can be identified: flares of pre‐existing dermatoses and new onset reactions.^3^

Autoimmune bullous reactions due to COVID 19 vaccines are not common, and to date only few cases following the first or second dose have been reported. To our knowledge, our patients represent the first patients developing a BP after the third dose of COVID‐19 vaccination. Interestingly, we did not observe any flare of bullous disease after the first or second dose of anti‐COVID‐19 vaccination.^4^

The pathogenesis should be explained by molecular mimicry between basement membrane‐specific proteins (e.g., BP‐180 and BP‐230) and SARS‐CoV‐2 spike protein, that is used by the virus to bind and fuse with host cells.^5^ Some authors however suggested that the vaccine may induce a great immunological response in individuals with immunological predisposition and consequent production of antibodies^6^; this explanation match with the two cases observed in patients not previously affected by bullous diseases.

In our experience, clinical presentation and management of vaccine induced BP fulfilled the diagnostic criteria of the classic form, but some cases reported in literature seem to suggest that atypical forms of BP may exist. Anyway, due to the lack of data, further studies are required to confirm this theory.^7^

As most of the population is vaccinated, clinicians should be aware that BP may occur or reactivate after COVID‐19 vaccination, especially in elderly patients. Given the rarity of these adverse events, clinicians should encourage the vaccination in patients affected by autoimmune bullous diseases, who may have a greater risk of be infected with SARS‐CoV‐2 compared with healthy individuals.^8^

## CONFLICT OF INTEREST

The authors declare no potential conflict of interest.

## AUTHOR CONTRIBUTIONS

All authors take public responsibility for the content of the work submitted for review; Federico Bardazzi, Federica Filippi, and Miriam Anna Carpanese contributed to conception of the study. Diego Abbenante, Federica Filippi, and Lidia Sacchelli contributed to acquisition and analysis of data.

## INFORMED CONSENT

Subjects have given their informed consent and the study protocol has been approved by the institute's committee on human research.

## Data Availability

The data that support the findings of this study are available from the corresponding author upon reasonable request.
